# Post-operative gamma knife radiosurgery for WHO grade I intracranial meningiomas: A single-center, retrospective study

**DOI:** 10.3389/fneur.2023.1094032

**Published:** 2023-02-13

**Authors:** Jinxiu Yu, Jiamin Zeng, Guanye Hu, Jing Wang, Guohao Chen, Minyi Huang, Shunyao Liang, Yong He, Yinhui Deng, Ye Gong, Junyi Fu

**Affiliations:** ^1^Department of Neurosurgery, Huashan Hospital, Fudan University, Shanghai, China; ^2^Department of Radiotherapy, The Second Affiliated Hospital of Guangzhou Medical University, Guangzhou, Guangdong, China; ^3^Department of Pathology, The Second Affiliated Hospital of Guangzhou Medical University, Guangzhou, Guangdong, China; ^4^Department of Neurology, The Second Affiliated Hospital of Guangzhou Medical University, Guangzhou, Guangdong, China

**Keywords:** stereotactic radiosurgery, gamma knife, meningioma, post-operative radiosurgery, malignant transformation

## Abstract

**Objective:**

To explore the results of the Gamma Knife radiosurgery (GKRS) for World Health Organization (WHO) grade I intracranial meningiomas after surgical resection.

**Methods:**

A total of 130 patients who were pathologically diagnosed as having WHO grade I meningiomas and who underwent post-operative GKRS were retrospectively reviewed in a single center.

**Results:**

Of the 130 patients, 51 patients (39.2%) presented with radiological tumor progression with a median follow-up time of 79.7 months (ranging from 24.0 to 291.3 months). The median time to radiological tumor progression was 73.4 months (ranging from 21.4 to 285.3 months), whereas 1-, 3-, 5-, and 10-year radiological progression-free survival (PFS) was 100, 90, 78, and 47%, respectively. Moreover, 36 patients (27.7%) presented with clinical tumor progression. Clinical PFS at 1, 3, 5, and 10 years was 96, 91, 84, and 67%, respectively. After GKRS, 25 patients (19.2%) developed adverse effects, including radiation-induced edema (*n* = 22). In a multivariate analysis, a tumor volume of ≥10 ml and falx/parasagittal/convexity/intraventricular location were significantly associated with radiological PFS [hazard ratio (HR) = 1.841, 95% confidence interval (CI) = 1.018–3.331, *p* = 0.044; HR = 1.761, 95% CI = 1.008–3.077, *p* = 0.047]. In a multivariate analysis, a tumor volume of ≥10 ml was associated with radiation-induced edema (HR = 2.418, 95% CI = 1.014–5.771, *p* = 0.047). Of patients who presented with radiological tumor progression, nine were diagnosed with malignant transformation. The median time to malignant transformation was 111.7 months (ranging from 35.0 to 177.2 months). Clinical PFS after repeat GKRS was 49 and 20% at 3 and 5 years, respectively. Secondary WHO grade II meningiomas were significantly associated with a shorter PFS (*p* = 0.026).

**Conclusions:**

Post-operative GKRS is a safe and effective treatment for WHO grade I intracranial meningiomas. Large tumor volume and falx/parasagittal/convexity/intraventricular location were associated with radiological tumor progression. Malignant transformation was one of the main cause of tumor progression in WHO grade I meningiomas after GKRS.

## Introduction

Meningiomas are benign intracranial tumors with the highest incidence rate, accounting for nearly 37.6% of all intracranial tumors (1). Almost 80.5% of the meningioma cases were confirmed by the World Health Organization (WHO) to be of grade I (1). However, the treatment for benign intracranial meningiomas remains challenging. The first treatment option is surgical resection (2). Some tumors, especially those located at favorable sites, can be radically treated by complete resection under certain conditions. However, for those located at unfavorable sites (such as those near neural or vascular structures and skull base tumors), it is difficult to perform complete resection as it can lead to severe complications.

Gamma Knife radiosurgery (GKRS), as a minimally invasive treatment method, is more attractive than general surgery. In a practical guideline (3), stereotactic radiosurgery (SRS) is recommended for residual, recurrent, or progressive meningiomas after surgical resection. Patients with WHO grade I meningiomas who underwent GKRS had 5- and 10-year progression-free survival (PFS) ranging from 85 to 100% and from 53 to 100%, respectively, with a low rate of adverse effects (3). However, the long-term tumor control rates of SRS vary widely among studies, and the causes of tumor progression remains unknown. Therefore, a single-center, retrospective study was conducted to explore the long-term results of post-operative GKRS for the WHO grade I intracranial meningiomas.

## Methods

### Patient selection

The medical records of patients with meningiomas who underwent GKRS in our center from December 1993 to December 2017 were retrospectively reviewed. In this study, a total of 130 patients who were pathologically diagnosed as having WHO grade I intracranial meningiomas underwent post-operative GKRS and had complete clinical data or had at least 24 months of follow-up were included. Furthermore, this study was approved by the institutional committee of the Second Affiliated Hospital of Guangzhou Medical University.

### Clinical and radiological evaluations

These patients were followed up regularly with clinical and radiological evaluations for the first 6 months and every year thereafter. Clinical tumor progression was defined as the development of new or worsening neurological signs or symptoms. Tumor volume shrinkage was defined as tumor volume of a shrinkage of at least 10%. Tumor volume change within 10% was deemed stable. Tumor progression was defined as an enlargement in tumor volume of at least 10% (4). Distant failure was defined as the formation of a new tumor far away from the prior irradiated tumor. The tumor volume was calculated using the formula: *V* = anteroposterior diameter × horizontal diameter × vertical diameter × π/6 (5).

### Radiosurgical techniques

The Elekta Leksell Gamma Knife instrument was used for GKRS treatment. The B-type gamma knife unit was used before April 2014, and the Perfexion unit was used from April 2014 to the present. After local anesthesia, Leksell stereotactic frame G was placed, and then, contrast magnetic resonance imaging (MRI) was performed to obtain tumor imaging for target delineation. Medical physicists, radiation oncologists, and neurosurgeons designed GKRS treatment plans. In this study, single-session GKRS was used for all patients.

### Statistical analysis

Univariate and multivariate analyses were used to identify the potential risk factors related to radiological and clinical PFS, as well as radiation-induced edema. Univariate and multivariate analyses were performed using log-rank test statistics and Cox proportional hazard models, respectively. The proportion of radiation-induced edema, as well as radiological and clinical PFS, was plotted using the Kaplan–Meier curves. Probability values of <0.05 were considered statistically significant. For statistical analysis, IBM SPSS version 26.0 was used.

## Results

### Baseline and treatment characteristics

The patient population consisted of 130 patients with WHO grade I intracranial meningiomas, including 48 men (36.9%) and 82 women (63.1%), with a median age of 47.8 years (ranging from 5.9 to 77.5 years). The median follow-up time was 79.7 months (ranging from 24.0 to 291.3 months). Of these patients, 27 patients (20.8%) presented with pre-existing peritumoral edema (PTE). There were 89 patients with symptomatic meningiomas (68.5%), including cranial nerve (CN) dysfunction (*n* = 45), headache (*n* = 53), dizziness (*n* = 35), seizures (*n* = 11), vomiting (*n* = 7), extremity weakness (*n* = 12), and ataxia (*n* = 6). All of the tumors had ever received surgical resection. Among them, 10 (7.7%) and 2 (1.5%) patients underwent surgery two or three times, respectively. The median time interval between surgery and GKRS was 6.7 months (ranging from 0.5 to 231.6 months). In this study, the residual (adjuvant SRS, *n* = 90) or post-operative progressive meningiomas (salvage SRS, *n* = 46) were indicators of post-operative GKRS for meningiomas. The median prescribed margin dose, the maximum dose, and isodose were 13.0, 33.0 Gy, and 40%, respectively. The median tumor volume was 8.6 ml (ranging from 0.1 to 55.6 ml). Additional characteristics are presented in Table 1.

**Table 1:** Baseline and treatment characteristics of 130 patients with intracranial WHO grade I meningiomas treated with post-operative GKRS.

**Characteristic**	***n* (%)**
No. of patients	130
Male, *n* (%)	48 (36.9)
Median age, (range), years	47.8 (5.9–77.5)
Median tumor volume at GKRS, (range), ml	8.6 (0.1–55.6)
Pre-existing PTE, *n* (%)	27 (20.8)
Median FU duration, (range), months	79.7 (24.0–291.3)
Median time interval between surgery and GKRS, (range), months	6.7 (0.5–231.6)
Adjuvant SRS, *n* (%)	90 (66.2)
Non-skull base tumors	51 (39.2)
No. of surgery, *n* (%)	118 (90.8)
Once	
Twice	10 (7.7)
Three times	2 (1.5)
Symptomatic tumors, *n* (%)	89 (68.5)
CN dysfunction	45 (34.6)
I	4 (3.1)
II	31 (23.8)
III/IV/VI	12 (9.2)
V	7 (5.4)
VII	3 (2.3)
VIII	9 (6.9)
Headache	53 (40.8)
Dizziness	35 (26.9)
Seizures	11 (8.5)
Vomiting	7 (5.4)
Extremity weakness	12 (9.2)
Ataxia	6 (4.6)
Tumor location, *n* (%)	
Foramen magnum	2 (1.5)
Frontobasal	5 (3.8)
Tentorium	9 (6.9)
Convexity	12 (9.2)
CPA	19 (14.6)
Falx/parasagittal	30 (23.1)
Intraventricular	7 (5.4)
Orbital	2 (1.5)
Parasellar/cavernous sinus	19 (14.6)
Petroclival	1 (0.7)
Sphenoidal	18 (13.8)
Suprasellar	6 (4.6)
Median margin dose, (range), Gy	13.0 (9.6-18.0)
Median maximum dose, (range), Gy	33.0 (21.6-50.0)
Median prescription isodose, (range), %	40 (25-60)

FU, follow up; GKRS, gamma knife radiosurgery; PTE, peritumoral edema; CPA, cerebellopontine angle; CN, cranial nerve.

### Radiological outcomes after GKRS

**Figure 1:**
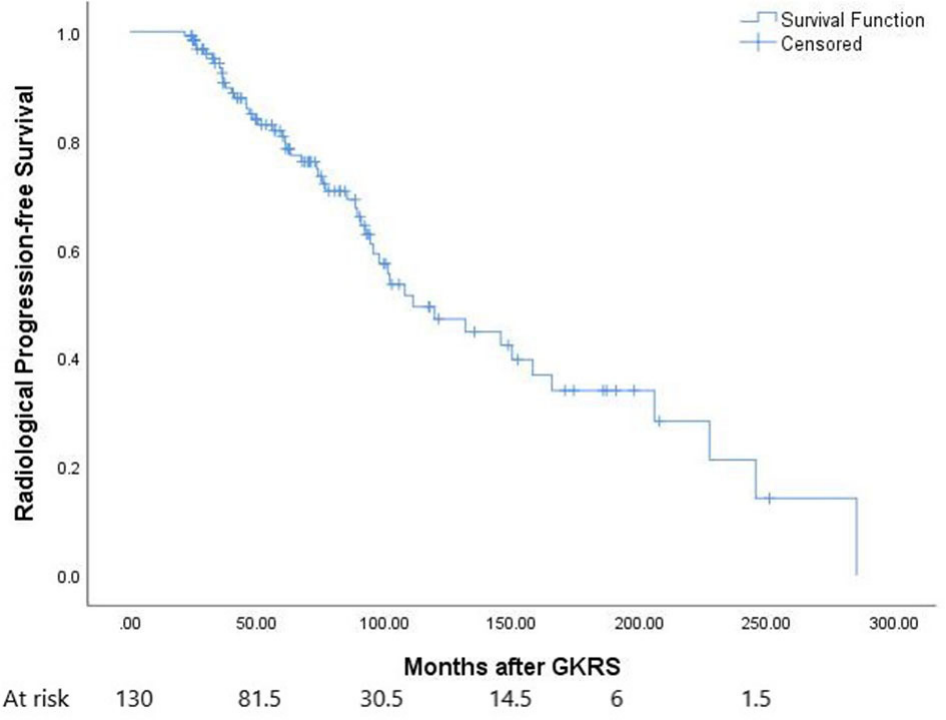
The Kaplan–Meier curve of radiological progression-free survival (PFS) in the entire series. The PFS was 100, 90, 78, and 47% at 1, 3, 5, and 10 years, respectively.

In this study, 51 patients (39.2%) presented with radiological tumor progression, 63 (48.5%) presented with tumor shrinkage, 16 (12.3%) presented with stable tumors, and 17 (13.1%) presented with distant failure (Table 2). The median time to radiological tumor progression was 73.4 months (ranging from 21.4 to 285.3 months), whereas 1-, 3-, 5-, and 10-year radiological PFS was 100, 90, 78, and 47%, respectively (Figure 1).

**Table 2:** GKRS treatment outcomes in the entire series.

**Outcomes**	***n* (%)**
**Radiological outcomes**	
Tumor control	79 (60.8)
Tumor shrinkage	63 (48.5)
Stable tumor	16 (12.3)
Progression	51 (39.2)
Distant failure	17 (13.1)
Clinical progression	36 (27.7)
CN dysfunction	11
I	1
II	7
III/IV/VI	2
V	2
VII	1
VIII	2
Headache	16
Seizures	8
Extremity numbness	2
Dizziness	7
Ataxia	2
Extremity weakness	4
Memory decline	1
GKRS related adverse effects	25 (19.2)
Radiation-induced edema	22 (16.9)
Symptomatic edema	11
CN II dysfunction	3

GKRS, gamma knife radiosurgery; CN, cranial nerve.

**Figure 2:**
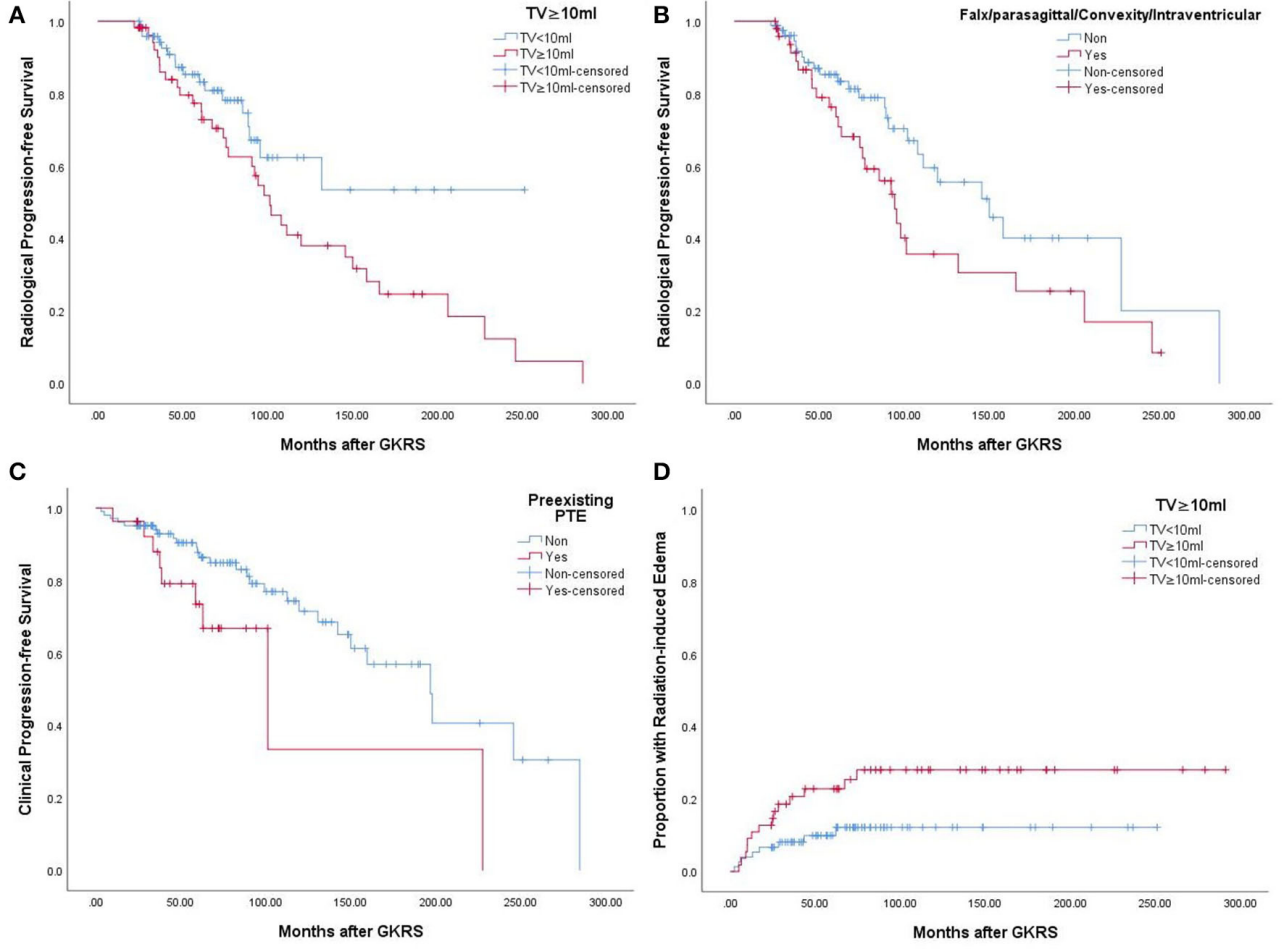
**(A)** Kaplan–Meier curve of radiological PFS of tumor volume ≥10 ml VS <10 ml in the entire series (*p* = 0.043). **(B)** Kaplan–Meier curve of radiological PFS of falx/parasagittal/convexity/intraventricular location in the entire series (*p* = 0.047). **(C)** Kaplan–Meier curve of clinical PFS of preexisting PTE in the entire series (*p* = 0.040). **(D)** Kaplan–Meier curve of radiation-induced edema of tumor volume ≥10 ml VS <10 ml in the entire series (*p* = 0.040).

According to the univariate analysis, a tumor volume of ≥ 10 ml (*p* = 0.043) (Figure 2A) and falx/parasagittal/convexity/intraventricular location (*p* = 0.047) (Figure 2B) were significantly associated with radiological tumor progression. In the multivariate analysis, a tumor volume of ≥10 ml and falx/parasagittal/convexity/intraventricular location were significantly associated with radiological PFS [hazard ratio (HR) = 1.841, 95% confidence interval (CI) =1.018–3.331, *p* = 0.044; HR = 1.761, 95% CI = 1.008–3.077, *p* = 0.047; Table 3].

**Table 3:** Univariate and multivariate cox proportional hazards regression analyses for radiological progression, radiation-induced edema, and clinical progression in the entire series.

**Characteristic**	**Radiological progression**				**Radiation-induced edema**				**Clinical progression**
	**Univariate**	**Multivariate**			**Univariate**	**Multivariate**			**Univariate**
	***p***	**HR**	**CI**	***P***	***p***	**HR**	**CI**	***P***	***p***
Age ≥ 55 years	0.944	NA	NA	NA	0.509	NA	NA	NA	0.183
Tumor volume ≥ 10 ml	**0.043^※^**	**1.841**	**1.018–3.331**	**0.044** ^※^	**0.040^※^**	**2.418**	**1.014–5.771**	**0.047^※^**	0.241
Falx/parasagittal/convexity/ intraventricular location	**0.047^※^**	**1.761**	**1.008–3.077**	**0.047^※^**	0.064	**NA**	**NA**	**0.069**	0.110
Pre-existing PTE	0.065	NA	NA	0.083	0.315	NA	NA	NA	**0.040^※^**
Adjuvant SRS	0.404	NA	NA	NA	0.399	NA	NA	NA	**0.667**
Symptomatic tumors	0.087	NA	NA	0.210	0.144	NA	NA	NA	0.403
Margin dose ≤ 13 Gy	0.914	NA	NA	NA	0.306	NA	NA	NA	0.200
Maximum dose ≤ 30 Gy	0.224	NA	NA	NA	0.389	NA	NA	NA	0.945
Post-operative residual	0.413	NA	NA	NA	0.399	NA	NA	NA	0.667

GKRS, gamma knife radiosurgery; PTE, peritumoral tumor edema; NA, not available; CI, confidential interval; HR, hazards ratio.

※ Statistically significant (P < 0.05).

Boldface type indicates statistical significance.

**Figure 3:**
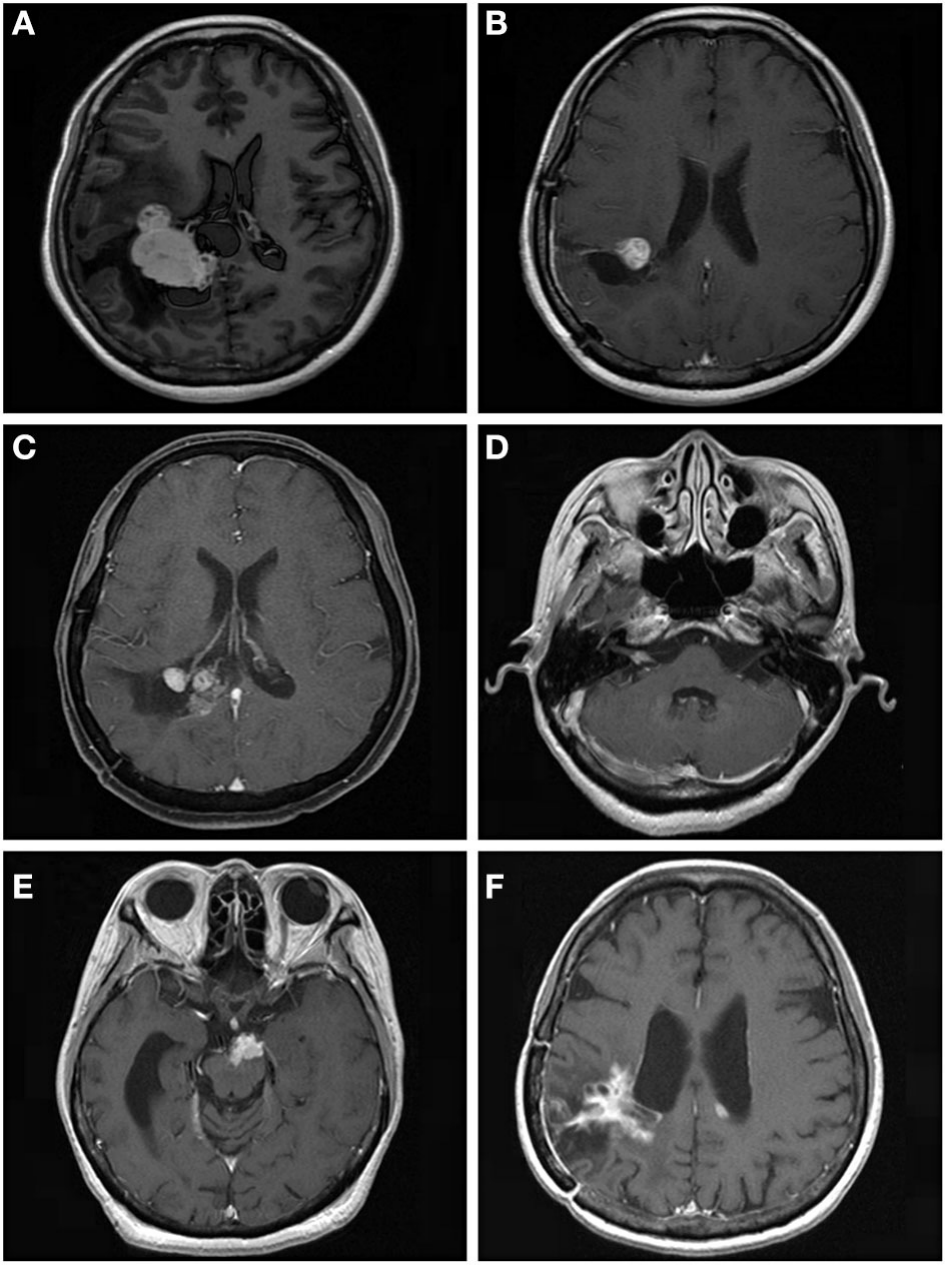
A 53.9-year-old woman with intraventricular World Health Organization (WHO) grade I meningioma received Gamma Knife radiosurgery (GKRS) (margin dose, 13.5 Gy; maximum dose, 30 Gy) as adjuvant treatment for residual tumor after surgical resection. The patient developed local tumor progression and intracranial implantation metastasis at 59.9 and 111.7 months, respectively. **(A)** contrast-enhanced T1-weighted magnetic resonance imaging (MRI) scans showed an intraventricular tumor. **(B)** Adjuvant GKRS for residual meningioma after surgical resection. **(C)** The patient developed local tumor progression at 59.9 months after GKRS. **(D–F)** The patient developed cerebellopontine angle, brainstem, and intraventricular metastasis at 111.7 months after surgical resection.

For further treatment of tumor progression, 29 patients underwent repeat GKRS, one patient underwent tomotherapy, seven patients died, and 14 patients underwent surgical resection with or without GKRS. Of the 14 patients, eight patients had malignant transformation with a histological diagnosis of WHO grade II. In addition, one patient with intracranial implantation metastasis 111.7 months after surgical resection was also considered to have malignant transformation (Figure 3). Finally, nine patients presented with malignant transformation. The median time to malignant transformation was 111.7 months (ranging from 35.0 to 177.2 months).

**Figure 4:**
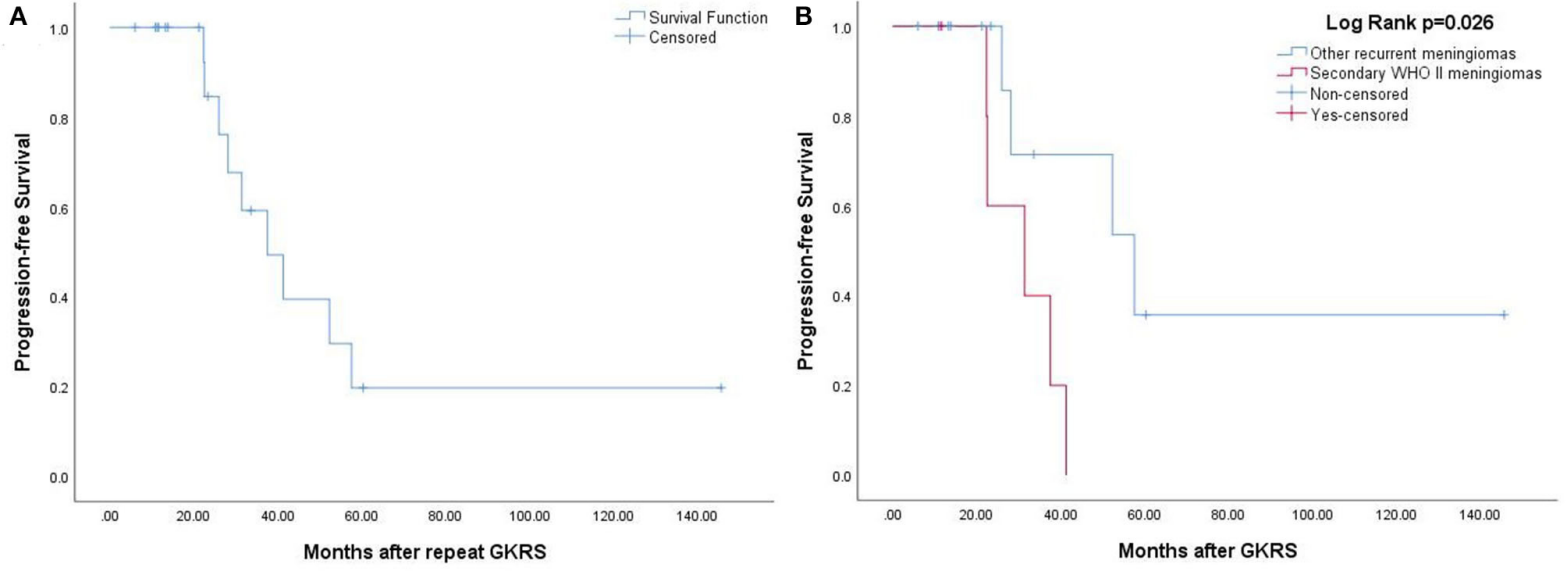
**(A)** The Kaplan–Meier curve of radiological PFS in 20 patients treated with repeat GKRS. The PFS was 100, 49, and 20% at 1, 3, and 5 years. **(B)** The Kaplan–Meier curve of radiological PFS of secondary WHO grade II meningiomas vs. other recurrent meningiomas (*p* = 0.026).

In total, 37 patients underwent repeat GKRS for tumor progression, with 17 patients missing. Of the remaining 20 patients undergoing repeat GKRS, six patients were diagnosed with secondary WHO grade II meningiomas. Finally, nine patients (45%) had tumor progression. The radiological PFS at 1, 3, and 5 years was 100, 49, and 20%, respectively (Figure 4A). Patients with secondary WHO grade II meningiomas were significantly associated with a shorter PFS in accordance with the Kaplan–Meier survival analysis (*p* = 0.026, Figure 4B).

### Clinical outcomes after GKRS

**Figure 5:**
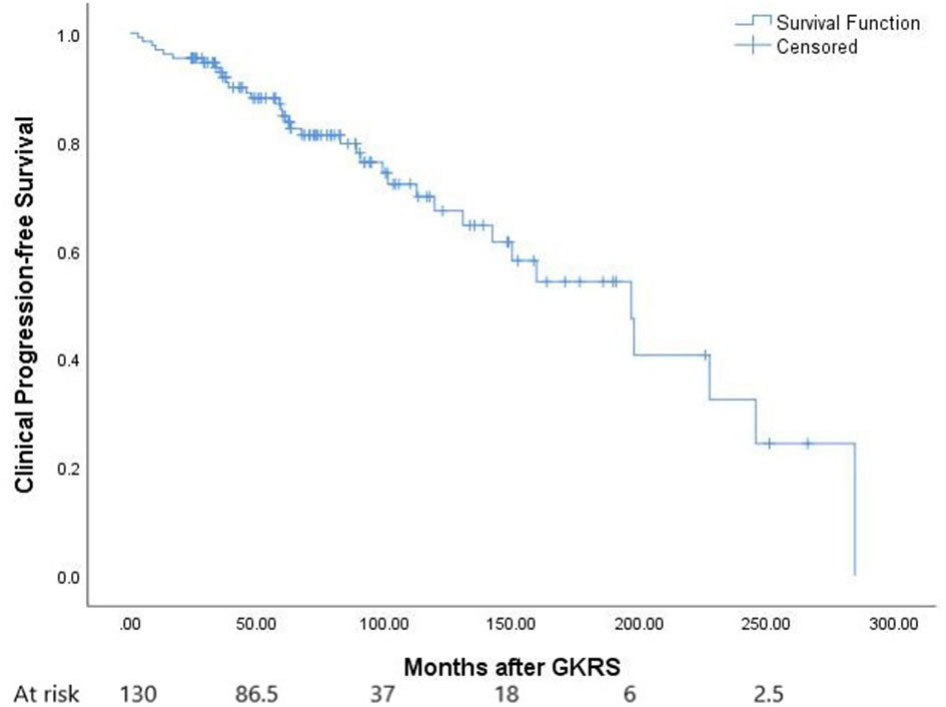
The Kaplan–Meier curve of clinical PFS in the entire series. Clinical PFS was 96, 91, 84, and 67% at 1, 3, 5, and 10 years.

After GKRS, 36 patients (27.7%) presented with clinical tumor progression, including CN dysfunction (*n* = 11), headache (*n* = 16), dizziness (*n* = 7), seizures (*n* = 8), extremity numbness (*n* = 2), extremity weakness (*n* = 4), memory decline (*n* = 1), and ataxia (*n* = 2) (Table 2). The median time to clinical tumor progression after GKRS was 62.0 months (ranging from 3.1 to 284.7 months). Of the 36 patients, the deterioration of neurological signs or symptoms in 23 patients were ascribable to tumor progression, 11 were ascribable to GKRS-related adverse effects, and two were due to distant failure. Clinical PFS at 1, 3, 5, and 10 years was 96, 91, 84, and 67%, respectively (Figure 5). According to the univariate analysis, only pre-existing PTE was found to be significantly associated with clinical PFS (*p* = 0.040, Figure 2C).

### GKRS-related adverse effects

**Figure 6:**
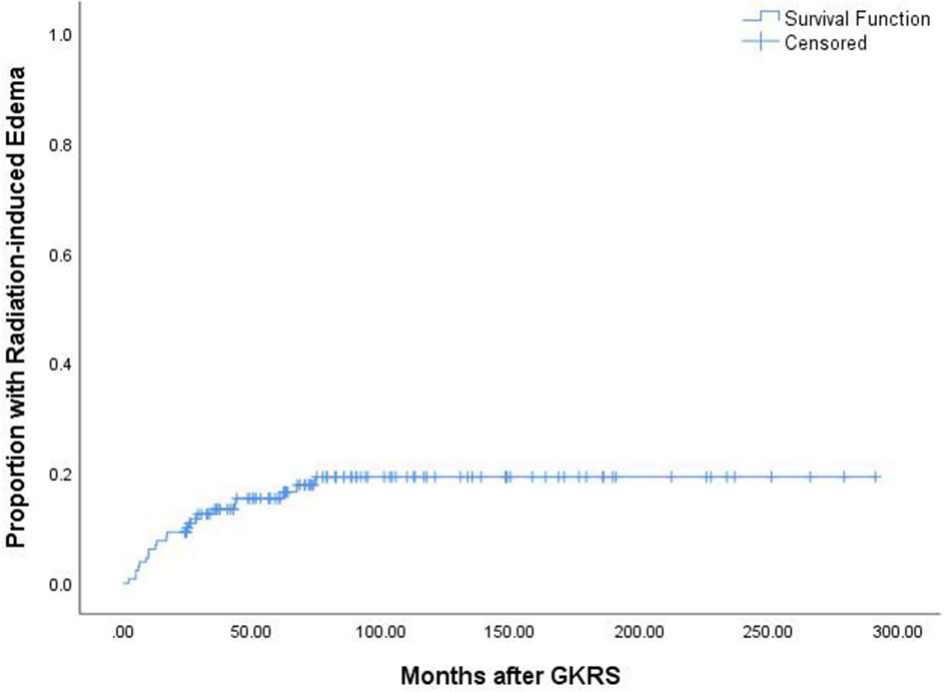
The Kaplan–Meier curve of proportion with radiation-induced edema in the entire series.

A total of 25 patients (19.2%) experienced GKRS-related adverse effects, including radiation-induced edema (*n* = 22) and CN II dysfunction (*n* = 3). The median time to these adverse effects was 17.1 months (ranging from 2.3 to 74.4 months). The adverse effect with the highest incidence rate in the current study was radiation-induced edema (Figure 6). Of the 22 patients who had radiation-induced edema complications, nine patients with symptomatic edema were relieved by corticosteroids, two patients presented with necrosis and severe edema and underwent surgical resection, and 11 patients with asymptomatic edema were kept under observation (Table 2).

According to the univariate analysis, a tumor volume of ≥10 ml was significantly associated with radiation-induced edema (*p* = 0.040, Figure 2D). The multivariate analysis showed that only a tumor volume of ≥10 ml was associated with radiation-induced edema (HR = 2.418, 95% CI = 1.014–5.771, *p* = 0.047) but not falx/parasagittal/convexity/intraventricular location (Table 3).

## Discussion

Surgery is still the cornerstone in the management of meningiomas. The European Association of Neuro-Oncology (EANO) guidelines recommend that surgical resection should be the first choice when treating meningiomas regardless of WHO grade (2). Surgery provides the advantages of quick tumor removal, rapid symptom relief, and precise pathological diagnosis. It does, however, rely on tumor sites and can cause significant mortality and morbidity. When tumors are located at unfavorable sites, complete resection is difficult. In this case, post-operative GKRS is needed.

### Tumor control after GKRS and related factors

Previous studies showed that the 5- and 10-year median PFS of benign meningiomas treated with SRS was 89% (ranging from 85 to 100%) and 85% (ranging from 53 to 100%), respectively (3). However, the long-term tumor control rate of patients with grade I meningiomas who underwent SRS varies widely between different studies. Seo et al. (6) reported that, after a median follow-up duration of 92 months in 424 patients with benign meningiomas treated with GKRS, the 5- and 10-year tumor control rates were 91.7 and 78.9%, respectively, and being female and having a history of craniotomy were associated with tumor progression. Hasegawa et al. (7) revealed that, in 67 patients with meningiomas treated with SRS, with a median follow-up time of 52 months (ranging from 7 to 195 months) and a median tumor volume of 4.9 ml (ranging from 0.7 to 22.9 ml), the 5- and 10-year actuarial local tumor control rates were 86 and 72%, respectively, and parasagittal/falcine location and previous surgery were significantly associated with failed tumor control. Starke et al. (8) showed that, in 75 patients with skull base meningiomas (>8 cm^3^) treated with SRS, the 5- and 10-year PFS was 88.6 and 77.2%, respectively, and a history of radiotherapy, TV > 14 cm^3^, and presentation with any CN deficit were associated with tumor progression. Santacroce et al. (9) indicated that, in 4,565 patients with benign meningiomas from 15 participating centers treated with SRS, the 5- and 10-year PFS was 95.2 and 88.6%, respectively, and the tumor control rate was higher for female, sporadic meningiomas, imaging-defined tumors, and skull base tumors. Azar et al. (10) revealed that, in 122 patients with meningioma who underwent GKRS, the 5-year PFS was 56.6%, with lower tumor volume and younger age being the factors related to PFS. Hasegawa et al. (11) found that, in 125 patients with convexity, parasagittal, or falcine meningiomas who underwent GKRS with a median tumor volume of 8 cm^3^ and a median follow-up time of 72 months, the 5- and 10-year actuarial PFS was 78 and 55%, respectively. In other studies (5, 12, 13), it was found that having prescribed a margin dose, being male, tumor sites (falx/parasagittal/convexity regions), and pre-GKRS KPS score were associated with tumor progression.

In this study, the median tumor volume was 8.6 ml. The 5- and 10-year PFS was 78 and 47%, respectively, which was lower than the studies of Hasegawa et al. (7) and Starke et al. (8) but higher than the study of Azar et al. (10). The lower tumor control rate in this study might be ascribable to the larger tumor volume and longer follow-up. Falx/parasagittal/convexity meningiomas are the most common non-skull base tumors, accounting for ~50–65% of intracranial meningiomas (14). A previous study reported that the falx/parasagittal/convexity regions were associated with tumor progression. Intraventricular meningiomas are rare non-skull base tumors. In this study, there were only seven cases of intraventricular meningiomas. All of them experienced tumor progression after GKRS, indicating a poor prognosis. As a result of the potential poor prognosis of falx/parasagittal/convexity meningiomas and intraventricular meningiomas, we decided to group falx, parasagittal, convexity, and intraventricular meningiomas into a single category. In the current study, a tumor volume of ≥10 ml and falx/parasagittal/convexity/intraventricular location were associated with radiological tumor progression.

Some studies explored multisession or staged SRS for large-volume meningiomas. Marchetti et al. (15) reported that 143 patients treated with multisession SRS received a median dose of 25 Gy in 3–5 fractions. The 5- and 8-year PFS was 93 and 90%, respectively, which was higher than the current study. However, the median follow-up time was only 44 months, which was insufficient. Iwai et al. (16) conducted research on 27 patients with large skull base meningiomas treated with staged GKRS. The median volume and diameter were 27.5 cm^3^ and 39.4 mm, respectively, whereas the 5-, 10-, and 15-year PFS was 78, 70, and 70%, respectively. Large meningiomas treated with hypofractionated stereotactic radiotherapy (HFRT) were also reported in several studies (17–20). Despite a small number of patients, some studies suggested that patients treated with HSRT might have a better tumor control rate. In a study by Han et al. (20), 70 patients with large meningiomas (>10 cm^3^) were treated with GKRS. At 5 years, the PFS rate in the HFRT group was higher than that in the single fraction group (92.9 vs. 88.1%), but there was no statistical significance (*p* = 0.389). In addition, the HFRT group had a lower complication rate (*p* = 0.017). In a study by Manabe et al. (18), the PFS of 5-fraction HFRT was similar to SRS. Therefore, the efficacy and safety of HFRT still remain unknown.

### Malignant transformation

It was estimated that 20–40% of meningiomas were secondary tumors (21–23). Numerous studies indicated that a stepwise genetic tumor progression contributing to a more malignant phenotype (24–26). As a consequence, WHO grade II or III meningiomas are classified as secondary or *de novo* tumors (27). A recent systematic review and meta-analysis indicated that the incidence rate of malignant transformation of WHO grade I meningiomas after surgery is 2.98/1,000 patient years. Skull base meningiomas have a lower incidence rate of malignant transformation than non-skull base tumors. Radiosurgery does not seem to increase the incidence rate of malignant transformation (28).

In the current study, only 14 out of 51 patients with WHO grade I meningiomas who presented with tumor progression after GKRS underwent surgical resection for further treatment. Finally, nine patients were diagnosed with malignant transformation, including eight patients with a pathological diagnosis of having WHO grade II and one patient with a clinical impression of intracranial implantation metastasis. The median time to malignant transformation was 111.7 months (ranging from 35.0 to 177.2 months). In this study, the malignant transformation was one of the main causes of tumor progression in WHO grade I meningiomas after GKRS. Therefore, when tumor progression in WHO grade I meningiomas occurs with the change of tumor biological behaviors after a number of years, attention should be paid to the issue of malignant transformation. Surgical resection is helpful for histological diagnosis, tumor removal, and subsequent multidisciplinary treatment. When progressive tumors are suspected of malignant transformation, surgical resection should be recommended as the first choice if it is appropriate.

### Clinical outcomes after GKRS and related factors

Previous studies indicated that the neurological deterioration rate ranges from 0 to 13.3% (median 7.4%) (3). Gupta et al. (29) reported 5- and 10-year actuarial symptom control rates were 86 and 70%, respectively, in 117 patients with asymptomatic meningiomas treated with GKRS. Seo et al. (6) found that 63 out of 424 patients experienced initial symptom aggravation, unfavorable outcomes, or new neurological deficits. Ge et al. (5) discovered that neurological signs or symptoms worsened in seven patients (5.4%) after GKRS and that pre-GKRS CN deficit and tumor volume of ≥10 ml were associated with the worsening of neurological signs or symptoms. In a study by Santacroce et al. (9), complications were observed in 12.9% of patients, and permanent grade 2 and 3 morbidity was 4.8%. In this study, 36 patients (27.7%) developed clinical tumor progression. Clinical PFS at 5 and 10 years was 84 and 67%, respectively. According to the univariate analysis, only pre-existing PTE was significantly associated with clinical PFS (*p* = 0.040). Compared to Ge's et al. (5) (27.7 vs. 5.4%), a higher rate of neurological signs or symptoms was observed in this study, which might be due to a higher rate of tumor progression, a larger tumor volume, and a longer follow-up period.

### Radiation-related adverse effects

According to a previous study (3), the median rate of SRS-related adverse effects was 8.0% (ranging from 2.5 to 34.6%). The adverse effect with the highest incidence rate is radiation-induced edema, accounting for 15–28% of all cases (30–37). A few factors such as sagittal sinus occlusion, parasagittal location, tumor volume, pre-existing PTE, hemispheric tumor location, and radiation doses have been reported to be associated with PTE (31–37). Seo et al. (6) found that 14% of patients had SRS-associated complications and 15% of patients experienced the development or aggravation of PTE after SRS. In multivariate analyses, skull base tumors, a history of craniotomy, and the presence of PTE before SRS were all significant and independent factors. Hasegawa et al. (7) discovered that 13.4% of patients developed mild or moderate adverse events after SRS. According to the univariate analysis, a higher margin dose was associated with adverse effects. In another study by Hasegawa et al. (11), the proportion of symptomatic PTE was higher in initial GKRS, and a lower margin dose and fewer prior treatments were associated with radiation-induced edema. Pollock et al. (12) showed that 11% of patients developed permanent radiation-related complications and that the parasagittal/flax/convexity location and tumor volume were associated with radiation-related complications. In the current study, one of the most common adverse effects was radiation-induced edema, and a tumor volume of ≥10 ml was significantly associated with radiation-induced edema.

### Study limitations

This study is a single-center, retrospective study. This study has several limitations that should be noted. First, in a retrospective study, treatment and selection biases were common. Second, because many patients underwent surgical resection in different hospitals, the pathological diagnosis was based on diagnostic criteria at the time. Third, of the 51 patients with meningioma having tumor progression, only 14 patients (27.5%) underwent surgical resection for further treatment, which might underestimate the proportion of malignant transformation. Fourth, the small number of patients in this study limited statistical power. Finally, due to the complex shapes after surgical resection, the formula used in this study to calculate tumor volume was only a rough estimate.

## Conclusions

In this study, post-operative GKRS was effective and safe for WHO grade I meningiomas, with a tumor control rate of 60.8%, a clinical tumor progression rate of 27.7%, and a GKRS-related adverse effect rate of 19.2%. Due to the many large tumors and long-term follow-up, the PFS rate at 10 years was 47%, which was relatively low in the current study. Thus, further studies are needed to improve the efficacy against large tumors. In addition, malignant transformation was found to be one of the main causes of tumor progression in WHO grade I meningiomas after GKRS. Therefore, more attention should be paid to the problem of malignant transformation when tumor progression occurs in WHO grade I meningiomas after GKRS.

## Data availability statement

The original contributions presented in the study are included in the article/supplementary material, further inquiries can be directed to the corresponding author.

## Ethics statement

This retrospective study was approved by the Institutional Committee of the Second Affiliated Hospital of Guangzhou Medical University. The patients/participants provided their written informed consent to participate in this study. Written informed consent was obtained from the individual(s) for the publication of any potentially identifiable images or data included in this article.

## Author contributions

JY, JF, JZ, MH, YH, SL, JW, GC, GH, and YD collected the data. JY and JZ analyzed the data. JY and JF wrote the paper. JF conceived and designed the study. YG directed and supervised the work. All authors agreed on the final paper.
